# Changes in dietary fat intake and associations with mental health in a UK public sample during the COVID-19 pandemic

**DOI:** 10.1093/pubmed/fdab009

**Published:** 2021-03-01

**Authors:** Jason J Wilson, Ilona McMullan, Nicole E Blackburn, Natalie Klempel, Anita Yakkundi, Nicola C Armstrong, Colette Brolly, Laurie T Butler, Yvonne Barnett, Louis Jacob, Ai Koyanagi, Lee Smith, Mark A Tully

**Affiliations:** Centre for Health and Rehabilitation Technologies, Institute of Nursing and Health Research, School of Health Sciences, Ulster University, Newtownabbey, BT37 0QB, UK; Institute of Mental Health Sciences, School of Health Sciences, Ulster University, Newtownabbey, BT37 0QB, UK; Sport and Exercise Sciences Research Institute, School of Sport, Ulster University, Newtownabbey, BT37 0QB, UK; Centre for Health and Rehabilitation Technologies, Institute of Nursing and Health Research, School of Health Sciences, Ulster University, Newtownabbey, BT37 0QB, UK; Institute of Mental Health Sciences, School of Health Sciences, Ulster University, Newtownabbey, BT37 0QB, UK; Centre for Health and Rehabilitation Technologies, Institute of Nursing and Health Research, School of Health Sciences, Ulster University, Newtownabbey, BT37 0QB, UK; Centre for Health and Rehabilitation Technologies, Institute of Nursing and Health Research, School of Health Sciences, Ulster University, Newtownabbey, BT37 0QB, UK; Institute of Mental Health Sciences, School of Health Sciences, Ulster University, Newtownabbey, BT37 0QB, UK; Northern Ireland Public Health Research Network, School of Health Sciences, Ulster University, Newtownabbey, BT37 0QB, UK; Health and Social Care Research & Development Division, Public Health Agency (Northern Ireland), Belfast, BT2 8BS, UK; Health and Social Care Health Improvement Division, Public Health Agency (Northern Ireland), Belfast, BT2 8BS, UK; School of Psychology and Sports Sciences, Anglia Ruskin University, Cambridge, CB1 1PT, UK; School of Life Sciences, Anglia Ruskin University, Cambridge, CB1 1PT, UK; Faculty of Medicine, University of Versailles Saint-Quentin-en-Yvelines, Montigny-le-Bretonneux 78180, France; Research and Development Unit, Parc Sanitari Sant Joan de Déu, CIBERSAM, Dr. Antoni Pujadas, 42, Sant Boi de Llobregat, Barcelona 08830, Spain; Research and Development Unit, Parc Sanitari Sant Joan de Déu, CIBERSAM, Dr. Antoni Pujadas, 42, Sant Boi de Llobregat, Barcelona 08830, Spain; ICREA, Pg. Lluis Companys 23, 08010 Barcelona, Spain; The Cambridge Centre for Sport and Exercise Sciences, Anglia Ruskin University, Cambridge, CB1 1PT, UK; Centre for Health and Rehabilitation Technologies, Institute of Nursing and Health Research, School of Health Sciences, Ulster University, Newtownabbey, BT37 0QB, UK; Institute of Mental Health Sciences, School of Health Sciences, Ulster University, Newtownabbey, BT37 0QB, UK

**Keywords:** COVID-19 pandemic, cross-sectional study, fat intake, mental health, social distancing

## Abstract

**Background:**

Consumption of unhealthy foods may have changed during the COVID-19 pandemic. This study explored how dietary fat intake was impacted in a sample of the UK public who were social distancing during the COVID-19 pandemic.

**Methods:**

Data were collected from a UK COVID-19 online survey. Fat intake was measured using the Dietary Instrument for Nutrition Education questionnaire. Anxiety and depressive symptoms were assessed using Becks’ Anxiety and Depression Inventories, while the short-form Warwick-Edinburgh Mental Well-being Scale assessed mental well-being. Differences between individuals who increased versus decreased fat intake were explored using chi-square or independent sample *t*-tests. Association between fat intake and mental health was explored using adjusted linear regression models.

**Results:**

Eight hundred and eighty-seven adults were included. Approximately, 34% recorded medium-to-high levels of fat consumption during social distancing. Around 48% reported decreased fat intake during social distancing compared to usual levels, while 41.3% documented increased fat intake. Fat intake was not significantly associated (*P* > 0.05) with any measures of mental health.

**Conclusions:**

A higher proportion of a sample of UK adults social distancing during the COVID-19 pandemic recorded decreased fat intake when compared to levels prior to social distancing. There appeared to be no associations between fat intake and mental health.

## Introduction

The worldwide spread of severe acute respiratory syndrome coronavirus 2 (i.e. SARS-CoV-2) was recognized as a global pandemic by the World Health Organization (WHO) in March 2020.^1^ As of 28 January 2020, the number of worldwide confirmed cases had reached 100 957 112, which had resulted in 2 177 045 deaths.^2^ COVID-19 is a particularly infectious coronavirus thought to spread mainly through respiratory droplets discharged when sneezing and coughing and through direct contact with contaminated surfaces.^3^ Due to the threat posed to public health, the UK Government, like many other countries, enforced lockdown restrictions which took effect on 26 March 2020. To reduce the risk of transmission, individuals were encouraged to stay at home and ‘socially distance’ throughout the lockdown period. Social distancing involves avoiding close contact with anyone not living in the same household. Except for limited essential purposes including: (i) shopping for food and medicine; (ii) medical needs, or providing care to others; (iii) one daily outdoor exercise such as walking, jogging or cycling alone or with members within the same household and (iv) travelling to a workplace when home-based working was not possible.^4^ Before national restrictions came into force, vulnerable individuals likely to suffer from serious complications from contracting COVID-19, such as those aged over 70 years old, those with underlying health conditions such as severe respiratory disease and chronic heart disease and also people who lived with vulnerable individual/s, were encouraged to stay at home.^4^

Although strategies such as social distancing are essential to control the spread of COVID-19, the downside is that lengthy periods of social distancing are likely to cause many individuals to become more anxious, depressed, frustrated and have increased feelings of isolation.^5^ A study of 932 UK adults social distancing due to COVID-19 found almost 37% experiencing poor mental health measured as moderate-to-severe anxiety symptoms, moderate-to-severe depressive symptoms and/or poor mental well-being.^6^ This study also highlighted that people who were younger, female, had lower annual incomes, smoked and had physical multimorbidity reported suffering from poorer mental health. Studies in other countries such as Brazil^7^ and China^8^ have also found similar negative impacts on anxiety and depressive symptoms.

This negative impact on mental health is likely to be compounded by unfavourable changes in health behaviours such as less active living and unhealthier eating. Feeling more anxious, depressed or stressed during the restriction period may lead to the adoption of ‘comfort eating’ behaviour, involving the consumption of high-fat or high-calorie foods.^9^^,^^10^ Increased fat intake in the long term, combined with reduced physical activity, is likely to lead to increased weight gain, and in turn, an increased risk of chronic conditions such as obesity, cardiovascular disease, type 2 diabetes and certain cancers.^9^ Early results published from the ECLB-COVID19 International Online Survey, which included 1047 participants, showed that compared to pre-self-isolation levels, there was an increased consumption of unhealthy foods, out of control eating and snacking during social distancing.^11^

A number of mechanisms have been proposed explaining why fat intake is likely to be associated with mental health. Higher saturated fat intake over a 4-year period has been shown to negatively influence global cognitive trajectories, possibly due to disruption of anti-inflammatory pathways typically observed in individuals with mental health illness.^12^^,^^13^ Unhealthy diets high in fat typically contain lower varieties of vitamins, minerals and fibres, which means lower intakes of important polyphenols. Polyphenols are important in controlling pathways related to neurogenesis and neuroprotection, which may lead to improvements in mental health symptoms such as depression.^12^ Processed foods typically contain high amounts of saturated fatty acids, artificial sweeteners and emulsifiers, which can negatively impact the bacteria within the gut, subsequently leading to inflammatory pathways being activated which can negatively impact brain health.^12^ Moderator variables, such as socio-economic status, are also likely to have an important influence on both diet and mental health.^14^^,^^15^ For example, low socio-economic status is likely to lead to a less healthy diet due to reduced access to healthy foods and poorer mental health due to social and environmental factors.^15^ Sub-group analysis from a review focusing on the impacts of dietary improvement interventions on mental health found that interventions which aimed to reduce fat intake had small beneficial effects on depression.^12^ It is important to note that associations are likely to be bi-directional, in that, poor mental health may lead to increased fat intake and vice versa.^15^ However, the associations between fat intake and mental health during social distancing are less clear. Research in this area is necessary as individuals with increased fat intake during social distancing may require support from interventions focusing on both healthy behaviour, such as nutrition and physical activity, and mental health improvement. This is particularly relevant due to the threat posed of further lockdowns due to second waves of the infection.

Therefore, the main aim of this study was to understand how fat intake was impacted in a sample of the UK public who were social distancing during the COVID-19 pandemic and also whether there were cross-sectional associations between fat intake and mental health, including anxiety symptoms, depressive symptoms and mental well-being. The hypotheses were that the majority of individuals will have increased their fat intake during social distancing and also that worse anxiety and depressive symptoms along with lower mental well-being will be associated with higher fat intake.

## Methods

### Design and participants

An online survey was launched in the UK on 17 March 2020. This study adopts a cross-sectional design presenting pre-planned interim analysis of data. Ethical approval was provided by the Anglia Ruskin University Research Ethics Committee on 16 March 2020.

Social media and national media outlets were used as the main methods of recruitment alongside invitations distributed through existing researcher networks. Inclusion criteria included: adults aged ≥18 years old, currently living in the UK and social distancing due to UK government-enforced restrictions to reduce the spread of COVID-19. Participants provided their informed consent after reading an information sheet using a data-encrypted website. Before completing the survey, individuals were asked two screener questions to confirm if they were currently aged ≥18 years old and were socially distancing. Prospective participants were only able to complete the survey by answering ‘Yes’ to both screener questions.

### Exposure variable

The Dietary Instrument for Nutrition Education (DINE) questionnaire was used to measure fat intake and has been validated for use in primary care.^16^^,^^17^ DINE measures consumption across 19 food groups over the previous week. Higher scores indicate higher fat intake. DINE classifies fat intake as ‘low’ (<30), ‘medium’ (30–40) or ‘high’ (>40). Low categorization represented an intake of ≤83 g/day, and high categorization represented an intake of >122 g/day.^16^ Participants filled in two DINE questionnaires; one for their usual fat intake before the COVID-19 pandemic and one for their fat intake while social distancing. This allowed the exploration of changes in fat intake which may have occurred.

### Outcome variables

Anxiety symptoms were measured using the Becks Anxiety Inventory (BAI), while depressive symptoms were measured using Becks Depression Inventory (BDI). Both questionnaires included 21 items, with higher BAI and BDI scores signifying worse anxiety and depressive symptoms. Both BAI and BDI have previously been deemed reliable and valid for use.^18^^,^^19^ The short-form Warwick-Edinburgh Mental Well-being Scale (SWEMWBS) was used to measure mental well-being. It contains seven items, with higher scores reflecting better mental well-being. It has been validated for use in research.^20^ Thresholds included: BAI score ≥16 for moderate-to-severe anxiety symptoms,^21^ BDI score ≥20 for moderate-to-severe depressive symptoms^22^ and SWEMWBS metric score ≤15.8 for poor mental well-being.^23^

### Covariates

The following demographic data were collected: age grouping (18–24, 25–34, 35–44, 45–54, 55–64 or ≥65 years old); gender (male, female or other); country (England, Scotland, Wales or Northern Ireland); annual household income (<£15 000, £15 000–24 999, £25 000–39 999, £40 000–59 999 or ≥£60 000); current smoking status (yes or no); current alcohol drinker (yes or no); self-reported time spent in moderate–vigorous physical activity (MVPA) per day since social distancing (minutes) and the number of chronic physical conditions (0 conditions, 1 condition or ≥2 conditions). Two or more physical conditions represented multimorbidity as defined in previous research.^24^ Chronic physical diseases included obesity, hypertension, myocardial infarction, angina pectoris and other coronary diseases, other cardiac diseases, varicose veins of lower extremities, osteoarthritis, chronic neck pain, chronic low back pain, chronic allergy (excluding allergic asthma), chronic bronchitis, emphysema or chronic obstructive pulmonary disease (COPD), Type 1 diabetes, Type 2 diabetes, diabetic retinopathy, cataract, peptic ulcer disease, urinary incontinence or urine control problems, hypercholesterolemia, chronic skin disease, chronic constipation, liver cirrhosis and other hepatic disorders, stroke, chronic migraine and other. The number of days spent self-isolating was recorded from the survey.

### Statistical analyses

To understand how fat intake changed when social distancing during the COVID-19 pandemic, the DINE fat intake score during social distancing was subtracted from the usual DINE fat intake score. Positive change scores (≥+1) suggested increased fat intake, negative change scores (≤−1) suggested decreased fat intake, while zero change scores suggested no change in fat intake during social distancing. A sensitivity analysis was also completed to expand the ‘no change’ definition to include those decreasing or increasing their fat intake within ±0.1 SD (standard deviation) (i.e. 1 score unit). This meant change scores needed to be at least ≥+2 or ≤−2 to be classified as changes in fat intake. The proportion of individuals who increased, decreased or had no change in their fat intake when social distancing was calculated. Differences between individuals who increased or decreased their fat intake were explored further using chi-square (categorical variables) or independent sample *t*-tests (continuous variables). As the assumptions were met for hierarchical linear regression analysis, the cross-sectional association between daily fat intake score calculated from DINE during the period of COVID-19 lockdown restrictions and mental health outcome (anxiety symptoms, depressive symptoms and mental well-being) was explored. Model 1 included one of the three mental health outcomes (anxiety symptoms, depressive symptoms or mental well-being). Model 2 adjusted for the number of days spent self-isolating, age grouping, gender, country, annual household income, current smoking status, current alcohol drinker, current levels of MVPA and the number of chronic physical conditions plus Model 1. Regression results including full details on the models are presented in the Supplementary Materials. Adjusted logistic regression analysis, exploring associations of daily fat intake during the COVID-19 lockdown restriction phase using mental health outcome groupings (i.e. low versus moderate-to-severe scores for anxiety and depressive symptoms as well as poor versus average-to-high scores for mental well-being) were also explored. However, these provided similar findings to the linear regression analysis, so were not included in the final results. Analyses were conducted using SPSS Version 25 (IBM, NY) with data presented as mean ± SD unless otherwise stated. Statistical significance was set at *P* < 0.05.

## Results

From the original 989 participants who completed the survey, 887 participants provided data for all the relevant study variables and were included in the final analysis (Table 1). Almost 81% of the sample were adults <65 years old, with almost two-thirds being women; the majority reported their nationality as English, had an annual income >£25 000 and were current alcohol drinkers; 12% were current smokers and 69.7% reported suffering from at least one chronic condition. Self-reported MVPA equated to nearly 90 min/day, while the mean number of days spent self-isolating at the time of online data collection was around 10 days. In terms of poor mental health, 31.2% had moderate-to-severe anxiety symptoms, 19.8% had moderate-to-severe depressive symptoms and 12.1% were classified as having poor mental well-being.

**Table 1:** Sample demographic and health characteristics including measures of mental health (*n* = 887)

*Characteristics*	*Category*	*Number (%)/mean ± SD*
Age	18–24 years old	91 (10.26)
	25–34 years old	185 (20.86)
	35–44 years old	146 (16.46)
	45–54 years old	152 (17.14)
	55–64 years old	143 (16.12)
	≥65 years old	170 (19.17)
Gender	Male	309 (34.84)
	Female	568 (64.04)
	Other	10 (1.13)
Country	England	692 (78.02)
	Scotland	22 (2.48)
	Wales	10 (1.13)
	Northern Ireland	163 (18.38)
Annual household income	<£15,000	139 (15.67)
	£15 000–24 999	165 (18.60)
	£25 000–39 999	202 (22.77)
	£40 000–59 999	180 (20.29)
	≥£60 000	201 (22.66)
Current smoking status	Yes	106 (11.95)
	No	781 (88.05)
Current alcohol drinker	Yes	596 (67.19)
	No	291 (32.81)
Time spent in MVPA per day, minutes	Mean ± SD	89.83 ± 102.37
Number of chronic physical conditions	No condition	269 (30.33)
	1 condition	233 (26.27)
	≥2 conditions	385 (43.40)
Number of days spent self-isolating	Mean ± SD	9.58 ± 7.12
BAI score	Mean ± SD	12.24 ± 11.71
BAI category	Low	610 (68.77)
	Moderate–severe	277 (31.23)
BDI score	Mean ± SD	11.65 ± 10.31
BDI category	Low	711 (80.16)
	Moderate–severe	176 (19.84)
SWEMWBS score	Mean ± SD	22.37 ± 5.98
SWEMWBS category	Poor	107 (12.06)
	Average-to-high	780 (87.94)

BAI = Becks Anxiety Inventory; BDI = Becks Depression Inventory; MVPA = moderate-vigorous physical activity; SD = standard deviation; SWEMWBS = Short-form Warwick-Edinburgh Mental Well-being Scale.

DINE fat intake scores in Table 2 highlight that 33.6% of the sample were consuming medium-to-high levels of fat while social distancing. Around 48% of the sample decreased their fat intake during social distancing compared to usual levels, while 41.3% increased their fat intake. Table 3 highlights the differences in sample demographic and health characteristics in individuals who increased versus decreased their fat intake while social distancing compared to usual levels. A significantly higher proportion of females (8.8%; *P* = 0.036) increased their fat intake during social distancing than decreased their fat intake.

**Table 2:** Changes in fat intake during social distancing (*n* = 887)

*Characteristics*	*Category*	*Number (%)/mean ± SD*
Usual DINE fat intake score	Mean ± SD	26.97 ± 10.56
Social distancing DINE fat intake score	Mean ± SD	26.62 ± 11.39
Social distancing DINE fat intake category	Low	589 (66.40)
	Medium	191 (21.53)
	High	107 (12.06)
Change in DINE fat intake score	Increased when social distancing	366 (41.26)
	Decreased when social distancing	424 (47.80)
	No change	97 (10.94)

DINE = Dietary Instrument for Nutrition Education; SD = standard deviation.

**Table 3:** Differences in sample demographic and health characteristics in individuals who increased versus decreased fat intake (*n* = 790)

*Characteristics*	*Category*	*Increased fat intake (*n* = 366)*	*Decreased fat intake (*n* = 424)*
		*Number (%)/mean ± SD*	*Number (%)/mean ± SD*
Age	18–24 years old	42 (11.48)	42 (9.91)
	25–34 years old	83 (22.68)	84 (19.81)
	35–44 years old	57 (15.57)	70 (16.51)
	45–54 years old	77 (21.04)	67 (15.80)
	55–64 years old	57 (15.57)	74 (17.45)
	≥65 years old	50 (13.66)	87 (20.52)
Gender^*^	Male	109 (29.78)	162 (38.21)
	Female	254 (69.40)	257 (60.61)
	Other	3 (0.82)	5 (1.18)
Country	England	291 (79.51)	330 (77.83)
	Scotland	10 (2.73)	9 (2.12)
	Wales	1 (0.27)	8 (1.89)
	Northern Ireland	64 (17.49)	77 (18.16)
Annual household income	<£15 000	48 (13.11)	72 (16.98)
	£15 000–24 999	62 (16.94)	87 (20.52)
	£25 000–39 999	86 (23.50)	89 (20.99)
	£40 000–59 999	75 (20.49)	87 (20.52)
	≥£60 000	95 (25.96)	89 (20.99)
Current smoking status	Yes	47 (12.84)	50 (11.79)
	No	319 (87.16)	374 (88.21)
Current alcohol drinker	Yes	237 (64.75)	289 (68.16)
	No	129 (35.25)	135 (31.84)
Time spent in MVPA per day, minutes	Mean ± SD	89.94 ± 106.18	85.37 ± 97.03
Number of chronic physical conditions	No condition	111 (30.33)	133 (31.37)
	1 condition	104 (28.42)	116 (27.36)
	≥2 conditions	151 (41.26)	175 (41.27)
Number of days spent self-isolating	Mean ± SD	10.01 ± 9.22	9.16 ± 5.18
BAI score	Mean ± SD	12.55 ± 11.12	12.38 ± 12.08
BDI score	Mean ± SD	12.29 ± 10.37	11.67 ± 10.36
SWEMWBS score	Mean ± SD	22.36 ± 5.84	22.24 ± 5.93

BAI = Becks Anxiety Inventory; BDI = Becks Depression Inventory; MVPA = moderate-vigorous physical activity; SD = standard deviation; SWEMWBS = Short-form Warwick-Edinburgh Mental Well-being Scale.

^*^Significant difference between groups (*P* < 0.05).

In the sensitivity analysis, which expanded the definition of no change to those decreasing or increasing their fat intake within ±0.1 SD (i.e. 1 score unit), 40.1% (*n* = 356) of the sample decreased their fat intake, 34.5% (*n* = 306) increased their fat intake and 25.4% (*n* = 225) had no change in their fat intake during social distancing compared to usual levels. Supplementary Table S1 (see online supplementary material) showed that there were no significant differences for any of the sample demographic and health characteristics; gender became non-significant (*P* = 0.053).

The associations between fat intake and anxiety symptoms, depressive symptoms and mental well-being are presented in the Supplementary Tables S2–S4 (see online supplementary material). The final model, adjusted for all relevant covariates, explained 18% of the variability in BAI score (*F*(10, 876) = 21.0, *P* < 0.001), 20% of the variability in BDI score (*F*(10, 876) = 22.6, *P* < 0.001) and 14% of the variability in SWEMWBS score (*F*(10, 876) = 15.6, *P* < 0.001). However, fat intake was not significantly associated (*P* < 0.05) with any measure of mental health.

## Discussion

### Main findings of this study

This study found that a higher proportion of individuals had reduced their fat intake during the period of social distancing compared to their consumption pre-social distancing. However, a sizeable proportion (41.3%) of the sample had increased their fat intake during the COVID-19 pandemic. In addition, no associations between dietary fat intake and mental health were observed in a sample of the UK public practicing social distancing during the COVID-19 pandemic.

### What is already known on this topic

A higher proportion of individuals decreasing their fat intake during social distancing is supported in another COVID-19-related survey in the USA, which found that in general, consumption of unhealthy foods containing high fat and high sugars had actually decreased during the COVID-19 pandemic.^25^ However, the author highlights this decrease was less pronounced in certain populations, such as those with high levels of obesity. One potential reason for this decrease could be a lack of access to certain foods, with different factors such as job loss, delays in receiving benefits, inability to travel to the shops or simply not having enough money, all contributing to reduced food availability.^26^ Another potential reason could be that less takeaway restaurants were open due to restrictions, with the food served from these establishments tending to be less healthy than home-cooked foods.^27^ Although not using directly comparable variables, another survey in Poland reported that 43.5% of their sample were eating more and 51.8% were snacking more during lockdown restrictions, which is not too dissimilar to the current study.^28^ Research in Italy, initially one of the worst-affected countries, highlights that 42.5% of individuals reported an increased consumption of comfort eating foods such as desserts and confectionaries.^29^ A large survey using data collected via Noom (a behavioural change weight loss app) found that the consumption of fruit and vegetables (low in fat) decreased, while the consumption of red and processed meats (generally high in fat content) increased during COVID-19.^30^ In our study, higher proportions of females increased versus decreased their fat intake during social distancing. This appears to contrast with the findings from Mitchell and colleagues who found that more females decreased their intake of ‘high fat condiments’ during social distancing; more so than the males in the same sample.^30^

The lack of association between fat intake and mental health is partially supported in other research that has found no associations between high-fat dairy consumption and mental health measures, including anxiety and depression.^31^ However, our finding is generally not supported in other literature that has shown that increased anxiety and depression are associated with unhealthy eating behaviour; generally representing higher fat consumption.^7^^,^^10^ Using more subjective methods, research in Italy during the COVID-19 pandemic has highlighted that 42.7% of individuals have attributed elevated anxiety with increased comfort eating.^29^ In a sample of Spanish adults from the general population, it was found that eating a healthy/balanced diet was one of the best predictors of decreased symptoms of anxiety (odds ratio = 0.85; *P* = 0.002) and depressive symptoms (odds ratio = 0.76; *P* < 0.001).^32^ One reason for this lack of association could be due to the relatively short period that participants had been social distancing for when they filled in the survey (i.e. 10 days). It may take longer for potential changes in fat intake to occur and have potential negative impacts on mental health (or vice versa), meaning the findings should be interpreted with caution. Another reason could be that weight status may be an important variable to consider; this was unfortunately not available from the current survey. Those that are overweight or obese may display different associations compared to normal weight individuals.^25^

### What this study adds

To the best of our knowledge, this study represents one of the first to explore the changes in dietary fat intake in a sample from the UK during the COVID-19 pandemic. A sizeable proportion of individuals increasing their fat intake, coupled with high levels of self-reported anxiety and low levels of physical activity being previously measured within this same sample,^6^ may result in a significant future public health burden if not addressed quickly. Particularly, as being overweight/obese has long-term negative health implications as well as increased susceptibility to COVID-19.^33^

### Limitations of this study

The current study had limitations which must be considered. The analysis for associations between fat intake and mental health was cross-sectional in nature. The average self-isolation period was relatively short at 10 days, meaning the results should be interpreted with a degree of caution. The generalizability of the sample to the UK population might be reduced due to the higher numbers of females completing the survey and to few participants from Scotland and Wales taking part. Some of the sample may have been shielding rather than only socially distancing, meaning they could not leave their home for any reason; this information was not recorded. However, it is unlikely this would have impacted the current study as many of the sample were young and middle-aged adults without chronic physical conditions. Fat intake was also self-reported, which may have introduced self-report bias compared with using a more extensive food diary. Comfort eating is not just confined to high-fat intake, it also incorporates the consumption of high-sugar and heavily salted foods which were not measured in the current study.

## Conclusions

In a sample of UK adults social distancing during the COVID-19 pandemic, a higher proportion appeared to consume less fat during social distancing than increased their fat intake compared to usual levels. However, there were still high numbers of individuals who had increased their fat intake (41.3%). There also appeared to be no associations between fat intake and mental health. There may be opportunities to reinforce public health messages around the benefits of positive lifestyle choices, such as healthy eating and being physically active, which seemed to have renewed focus within the general public during the early stages of the COVID-19 pandemic. Future research should attempt to establish the characteristics of individuals who are likely to increase their fat intake during the COVID-19 pandemic as interventions may need to be developed in certain population groups to prevent negative eating habits being established in the long term. It would also be useful to explore other dietary components, such as sugar and salt intake, which may also have adverse impacts on mental health.

## Supplementary Material

Fat_and_mental_health_suppl_fdab009

## Notes

**Jason J. Wilson,** Lecturer in Physical Activity and Health

**Ilona McMullan,** Research Associate

**Nicole E. Blackburn,** Lecturer in Healthcare Science

**Natalie Klempel,** Research Assistant

**Anita Yakkundi,** Research Associate and Network Coordinator

**Nicola C. Armstrong,** Programme Manager

**Colette Brolly,** Health and Social Wellbeing Improvement Manager

**Laurie T. Butler,** Pro Vice-Chancellor and Dean

**Yvonne Barnett,** Deputy Vice-Chancellor for Research and Innovation

**Louis Jacob,** Resident in Physical Medicine and Rehabilitation

**Ai Koyanagi,** Research Professor

**Lee Smith,** Reader in Physical Activity and Public Health

**Mark A. Tully,** Professor of Public Health
