# Supplementary material for: Changes in dietary fat intake and associations with mental health in a UK public sample during the COVID-19 pandemic
Source: J Public Health (Oxf). 2021 Mar 1;43(4):687–94. doi: 10.1093/pubmed/fdab009 (PMC7989334; doi:10.1093/pubmed/fdab009)
Supplement: Fat_and_mental_health_suppl_fdab009 [file fat_and_mental_health_suppl_fdab009.docx]

***Supplementary Material (Tables S1-S4)***

Table S1: Differences in sample demographic and health characteristics in individuals who increased versus decreased fat intake (n=662)

| **Characteristics** | **Category** | **Increased fat intake (n=306)** | **Decreased fat intake (n=356)** |
| --- | --- | --- | --- |
|  |  | **Number (%) / Mean ± SD** | **Number (%) / Mean ± SD** |
| Age | 18-24 years old  25-34 years old  35-44 years old  45-54 years old  55-64 years old  ≥65 years old | 33 (10.78)  77 (25.16)  45 (14.71)  59 (19.28)  48 (15.69)  44 (14.38) | 35 (9.83)  72 (20.22)  63 (17.70)  51 (14.33)  63 (17.70)  72 (20.22) |
| Gender | Male  Female  Other | 91 (29.74)  214 (69.93)  1 (0.33) | 135 (37.92)  218 (61.24)  3 (0.84) |
| Country | England  Scotland  Wales  Northern Ireland | 244 (79.74)  8 (2.61)  0 (0.00)  54 (17.65) | 279 (78.37)  8 (2.25)  5 (1.40)  64 (17.98) |
| Annual household income | <£15,000  £15,000 - £24,999  £25,000 - £39,999  £40,000 - £59,999  ≥£60,000 | 44 (14.38)  53 (17.32)  69 (22.55)  62 (20.26)  78 (25.49) | 57 (16.01)  75 (21.07)  76 (21.35)  78 (21.91)  70 (19.66) |
| Current smoking status | Yes  No | 40 (13.07)  266 (86.93) | 42 (11.80)  314 (88.20) |
| Current alcohol drinker | Yes  No | 197 (64.38)  109 (35.62) | 237 (66.57)  119 (33.43) |
| Time spent in MVPA per day, minutes | Mean ± SD | 89.97 ± 106.82 | 81.87 ± 92.44 |
| Number of chronic physical conditions | No condition  1 condition  ≥2 conditions | 91 (29.74)  82 (26.80)  133 (43.46) | 123 (34.55)  90 (25.28)  143 (40.17) |
| Number of days spent self-isolating | Mean ± SD | 9.99 ± 9.73 | 9.13 ± 5.36 |
| BAI score | Mean ± SD | 12.64 ± 11.06 | 12.58 ± 11.88 |
| BDI score | Mean ± SD | 12.25 ± 10.13 | 12.15 ± 10.50 |
| SWEMWBS score | Mean ± SD | 22.49 ± 5.87 | 21.79 ± 5.93 |

Abbreviations: BAI = Becks Anxiety Inventory; BDI = Becks Depression Inventory; BMI = body mass index; MVPA; moderate-vigorous physical activity; SD = standard deviation; SWEMWBS = Short-form Warwick-Edinburgh Mental Well-being Scale

*Significant difference between groups (p<0.05).

***DINE fat score and BAI score***

Assumptions for linear regression were met. Consequently, a hierarchical linear regression was calculated using the BAI scores (Table S2). Separate multiple linear regressions, adjusted for MPVA and participant demographic and health characteristics, were developed to understand the association between DINE fat score and BAI score.

Model 1 showed that DINE fat score did not reliably predict BAI score (p=0.496). Model 2 included the effects of DINE fat score, and participant demographic and health covariates including the number of days spent self-isolating, age grouping, gender, country, income, current smoking status, current alcohol drinker, current levels of MVPA and the number of chronic physical conditions, and reliably predicted BAI score (F (10, 876)=20.74, p<0.001) where 18% of the variance was explained (R²=0.18).

The analysis of the fully adjusted model (Model 2) showed that DINE fat score (p=0.348) was not a significant independent predictor of anxiety symptoms measured using the BAI. Additionally, the covariates of age (β=-2.03, t (876)=-8.11, p<0.001); gender (β=4.49, t (876)=6.11, p<0.001); annual household income (β=-0.94, t (876)=-3.47, p=0.001); smoking (β=-6.09, t (876)=-5.38, p<0.001); and number of chronic physical conditions (β=2.46, t (876)=5.22, p<0.001) were also found to be statistically significant predictors of anxiety symptoms. Being older, male, higher household income, non-smoker as well as lower number of chronic physical conditions resulted in lower anxiety symptoms. Days of self-isolation (p=0.476), country (p=0.671), alcohol (p=0.442) and MVPA (p=0.089) were not found to be significantly associated with anxiety symptoms.

Table S2: Linear model of predictors of BAI scores including DINE fat intake scores with 95% Confidence Intervals

| Model | | Unstandardized Coefficients | | Standardized Coefficients | | T | | p | | 95.0% Confidence Interval for B | | |  |
| --- | --- | --- | --- | --- | --- | --- | --- | --- | --- | --- | --- | --- | --- |
|  |  | B | Std. Error | | Beta | |  | |  | | Lower Bound | Upper Bound | |
| 1 | (Constant) | 11.61 | 1.00 | |  | | 11.61 | | 0.000 | | 9.65 | 13.57 | |
|  | DINE fat score | 0.02 | 0.03 | | 0.02 | | 0.68 | | 0.496 | | -0.04 | 0.09 | |
| 2 | (Constant) | 22.20 | 3.00 | |  | | 7.40 | | 0.000 | | 16.31 | 28.10 | |
|  | DINE fat score | 0.03 | 0.03 | | 0.03 | | 0.94 | | 0.348 | | -0.03 | 0.09 | |
|  | Days in self-isolation | 0.04 | 0.05 | | 0.02 | | 0.71 | | 0.476 | | -0.06 | 0.14 | |
|  | Age | -2.03 | 0.25 | | -0.29 | | -8.11 | | 0.000 | | -2.52 | -1.54 | |
|  | Gender | 4.49 | 0.74 | | 0.19 | | 6.11 | | 0.000 | | 3.05 | 5.94 | |
|  | Country | 0.13 | 0.31 | | 0.01 | | 0.42 | | 0.671 | | -0.48 | 0.74 | |
|  | Annual household income | -0.94 | 0.27 | | -0.11 | | -3.47 | | 0.001 | | -1.47 | -0.41 | |
|  | Smoking | -6.09 | 1.13 | | -0.17 | | -5.38 | | 0.000 | | -8.31 | -3.86 | |
|  | Alcohol | 0.60 | 0.78 | | 0.02 | | 0.77 | | 0.442 | | -0.93 | 2.12 | |
|  | Average MVPA per day | -0.01 | 0.00 | | -0.05 | | -1.70 | | 0.089 | | -0.01 | 0.00 | |
|  | Chronic physical conditions | 2.46 | 0.47 | | .18 | | 5.22 | | 0.000 | | 1.54 | 3.39 | |

***DINE fat score and BDI score***

Assumptions for linear regression were met. Consequently, a hierarchical linear regression was calculated using the BDI scores (Table S3). Separate multiple linear regressions, adjusted for MPVA and participant demographic and health characteristics, were developed to understand the association between DINE fat score and BDI score.

Model 1 showed that DINE fat score did not reliably predict BDI score (p=0.378). Model 2 included the effects of DINE fat score, and participant demographic and health covariates including the number of days spent self-isolating, age grouping, gender, country, income, current smoking status, current alcohol drinker, current levels of MVPA and the number of chronic physical conditions, and reliably predicted BDI score (F (10, 876)=22.60, p<0.001) where 20% of the variance was explained (R²=0.20).

The analysis of the fully adjusted model (Model 2) showed that DINE fat score (p=0.274) was not a significant independent predictor of depressive symptoms measured using the BDI. Additionally, the covariates of age (β=-2.02, t (876)=-9.22, p<0.001); gender (β=1.74, t (876)=2.71, p=0.007); annual household income (β=-0.96, t (876)=-4.06, p<0.001); smoking (β=-5.43, t (876)=-5.49, p<0.001); MVPA (β=-0.01, t (876)=-3.73, p<0.001) and number of chronic physical conditions (β=2.31, t (876)=5.62, p<0.001) were also found to be statistically significant predictors of depressive symptoms. Being older, male, higher household income, non-smoker, higher time in MVPA as well as lower number of chronic physical conditions resulted in lower depressive symptoms. Days of self-isolation (p=0.262), country (p=0.643) and alcohol (p=0.316) were not found to be significantly associated with depressive symptoms.

Table S3: Linear model of predictors of BDI scores including DINE fat intake scores with 95% Confidence Intervals

| Model | | Unstandardized Coefficients | | Standardized Coefficients | t | p | 95.0% Confidence Interval for B | |
| --- | --- | --- | --- | --- | --- | --- | --- | --- |
|  |  | B | Std. Error | Beta |  |  | Lower Bound | Upper Bound |
| 1 | (Constant) | 10.94 | 0.88 |  | 12.42 | 0.000 | 9.21 | 12.66 |
|  | DINE fat score | 0.03 | 0.03 | 0.03 | 0.88 | 0.378 | -0.03 | 0.09 |
| 2 | (Constant) | 26.75 | 2.62 |  | 10.20 | 0.000 | 21.60 | 31.89 |
|  | DINE fat score | 0.03 | 0.03 | 0.03 | 1.09 | 0.274 | -0.02 | 0.08 |
|  | Days in self-isolation | -0.05 | 0.04 | -0.03 | -1.12 | 0.262 | -0.14 | 0.04 |
|  | Age | -2.02 | 0.22 | -0.32 | -9.22 | 0.000 | -2.44 | -1.59 |
|  | Gender | 1.74 | 0.64 | 0.08 | 2.71 | 0.007 | 0.48 | 3.00 |
|  | Country | -0.13 | 0.27 | -0.01 | -0.46 | 0.643 | -0.66 | 0.41 |
|  | Annual household income | -0.96 | 0.24 | -0.13 | -4.06 | 0.000 | -1.42 | -0.49 |
|  | Smoking | -5.43 | 0.99 | -0.17 | -5.49 | 0.000 | -7.37 | -3.49 |
|  | Alcohol | 0.68 | 0.68 | 0.03 | 1.00 | 0.316 | -0.65 | 2.01 |
|  | Average MVPA per day | -0.01 | 0.00 | -0.12 | -3.73 | 0.000 | -0.02 | -0.01 |
|  | Chronic physical conditions | 2.31 | 0.41 | 0.19 | 5.62 | 0.000 | 1.51 | 3.12 |

***DINE fat score and SWEMWBS***

Assumptions for linear regression were met. Consequently, a hierarchical linear regression was calculated using the SWEMWBS scores (Table S4). Separate multiple linear regressions, adjusted for MPVA and participant demographic and health characteristics, were developed to understand the association between DINE fat score and SWEMWBS score.

Model 1 showed that DINE fat score did not reliably predict SWEMWBS score (p=0.419). Model 2 included the effects of DINE fat score, and participant demographic and health covariates including the number of days spent self-isolating, age grouping, gender, country, income, current smoking status, current alcohol drinker, current levels of MVPA and the number of chronic physical conditions, and reliably predicted SWEMWBS score (F (10, 876)=15.87, p<0.001) where 14% of the variance was explained (R²=0.14).

The analysis of the fully adjusted model (Model 2) showed that DINE fat score (p=0.602) was not a significant independent predictor of anxiety symptoms measured using the SWEMWBS. Additionally, the covariates of age (β=1.16, t (876)=-8.86, p<0.001); annual household income (β=0.29, t (876)=2.06, p=0.040); smoking (β=2.20, t (876)=3.72, p<0.001); MVPA (β=0.01, t (876)=3.09, p=0.002) and number of chronic physical conditions (β=-0.96, t (876)=-3.90, p<0.001) were also found to be statistically significant predictors of mental wellbeing. Being older, male, higher household income, non-smoker, higher time in MVPA as well as lower number of chronic physical conditions resulted in better mental wellbeing. Days of self-isolation (p=0.210), gender (p=0.078), country (p=0.290), alcohol (p=0.552) were not found to be significantly associated with mental wellbeing.

Table S4: Linear model of predictors of SWEMWBS scores including DINE fat intake scores with 95% Confidence Intervals

| Model | | Unstandardized Coefficients | | Standardized Coefficients | t | p | 95.0% Confidence Interval for B | |
| --- | --- | --- | --- | --- | --- | --- | --- | --- |
|  |  | B | Std. Error | Beta |  |  | Lower Bound | Upper Bound |
| 1 | (Constant) | 21.99 | 0.51 |  | 43.06 | 0.000 | 20.99 | 22.99 |
|  | DINE fat score | 0.01 | 0.02 | 0.03 | 0.81 | 0.419 | -0.02 | 0.05 |
| 2 | (Constant) | 14.27 | 1.57 |  | 9.09 | 0.000 | 11.19 | 17.35 |
|  | DINE fat score | 0.01 | 0.02 | 0.02 | 0.52 | 0.602 | -0.02 | 0.04 |
|  | Days in self-isolation | 0.03 | 0.03 | 0.04 | 1.25 | 0.210 | -0.02 | 0.08 |
|  | Age | 1.16 | 0.13 | 0.32 | 8.86 | 0.000 | 0.90 | 1.42 |
|  | Gender | -0.68 | 0.38 | -0.06 | -1.76 | 0.078 | -1.43 | 0.08 |
|  | Country | 0.17 | 0.16 | 0.03 | 1.06 | 0.290 | -0.15 | 0.49 |
|  | Annual household income | 0.29 | 0.14 | 0.07 | 2.06 | 0.040 | 0.01 | 0.57 |
|  | Smoking | 2.20 | 0.59 | 0.12 | 3.72 | 0.000 | 1.04 | 3.36 |
|  | Alcohol | -0.24 | 0.41 | -0.02 | -0.59 | 0.552 | -1.04 | 0.56 |
|  | Average MVPA per day | 0.01 | 0.00 | 0.10 | 3.09 | 0.002 | 0.00 | 0.01 |
|  | Chronic physical conditions | -0.96 | 0.25 | -0.14 | -3.90 | 0.000 | -1.44 | -0.48 |
